# Unravelling sources of fecal pollution in oligotrophic mountain waters: Integrating *Escherichia coli* enumeration, microbial source tracking, and eDNA analysis

**DOI:** 10.1007/s10661-025-14298-7

**Published:** 2025-07-03

**Authors:** Sharon Maes, Martin Andersson-Li, Jessica Sjöstedt, Jon Hildahl, Daniel Yu, Norman Neumann, Monica Odlare, Anders Jonsson

**Affiliations:** 1 Faculty of Science, Technology and Media, Department of Natural Science, Design and Sustainable Development, Mid Sweden University, Akademigatan 1, SE-831 25 Östersund, Sweden; 2 AquaBiota Water Research ABWR AB, Sveavägen 159, SE-113 46 Stockholm, Sweden; 3 MoRe Research Örnsköldsvik AB, Box 70, SE-891 22 Örnsköldsvik, Sweden; 4 Hjortens Laboratorium, Hyggesvägen 21B, SE-831 48 Östersund, Sweden; 5 School of Public Health, University of Alberta, 11328 - 89 Ave NW, Edmonton, AB Canada; 6 Present Address: Calluna AB, Hästholmsvägen 28, SE-131 30 Nacka, Sweden

**Keywords:** *E. coli*, eDNA, Fecal contamination, Microbial Source Tracking

## Abstract

Aquatic ecosystems in mountainous regions are crucial for fulfilling natural and anthropogenic water demands around the world. This study integrates *Escherichia coli* (*E. coli*) enumeration, microbial source tracking (MST), and environmental DNA (eDNA) analysis to identify sources of fecal contamination in oligotrophic mountain waters. Conducted in an area with intense tourism and traditional reindeer herding, this research addresses the urgent need to identify fecal pollution sources to safeguard the water quality of these vital ecosystems. Our study reveals that *E. coli* levels vary significantly across different locations and times, suggesting varied sources of contamination from humans, wildlife, and livestock animals. MST techniques, alongside eDNA analysis, provided insights into the complex patterns of fecal pollution, allowing for the distinction between human and animal contributions to water contamination. Our findings highlight the importance of combining various analytical methods to track fecal pollution sources effectively, and to develop targeted strategies for water quality management.

**Supplementary Information:**

The online version contains supplementary material available at 10.1007/s10661-025-14298-7.

## Introduction

Aquatic ecosystems in mountain areas worldwide play a critical role in meeting natural and anthropogenic water demands. In recognition of their immense importance, mountains have been called the “Water Towers” of the world, providing freshwater to a substantial part of the world's population (Immerzeel et al., 2020; Messerli et al., 2004). These freshwater mountain ecosystems, however, are under significant threat (Vári et al., 2022), while providing essential ecosystem resources and safe drinking water for their surrounding and downstream rural and urban communities. The Intergovernmental Panel on Climate Change (IPCC) has highlighted alpine regions as climate change hotspots (IPCC, 2018) and it is projected that overshooting global temperatures by more than 1.5 °C will result in irreversible adverse impacts on certain low-resilience ecosystems (IPCC, 2023). Increasing and diversifying tourism in these regions also exerts pressure (e.g., through the increase of diffuse fecal pollution by campers hiking in the terrain and increasing pressure on local sewage systems at tourist facilities) on these areas and their sensitive aquatic ecosystems (Evander & Ahlm, 2009; Maes et al., 2022). Indeed, the degradation of water quality due to fecal pollution can have severe negative ecosystem impacts, which can in turn affect the surrounding wildlife, visitors, and inhabitants, as well as the Indigenous communities that depend on the land for their daily income. In light of this, the major importance of clean drinking water to human health has been recognised through Sustainable Development Goal 6.1, which aims to: “*Ensure availability and sustainable management of water and sanitation for all*” (United Nations, 2015).

Since many transmissible pathogens are present in the feces of warm-blooded organisms, fecal pollution has been recognized as a major cause of waterborne diseases (Leclerc et al., 2002). *Escherichia coli* (*E. coli*) is often used as an indicator of fresh fecal pollution (and accordingly pathogens) in water (Holcomb & Stewart, 2020) because of its ubiquitous presence in the lower intestine of warm-blooded animals (Savageau, 1983), and the availability of reliable and quick methods for its detection. If *E. coli* is of human origin, it can also be an indicator of anthropogenic contaminants, including pharmaceuticals, personal care products, and endocrine-disrupting chemicals with direct or indirect impacts on human and environmental health (Paruch & Paruch, 2021). Additionally, *E. coli* is also considered an important carrier of antibiotic resistance genes in freshwater river systems, especially from anthropogenic sources (Ågerstrand et al., 2023; Bong et al., 2022).

To limit or prevent the discharge of fecal contamination into rivers, it is important to know the location and source of pollution. Structured water sampling alone, however, may fail to pinpoint the sources of fecal pollution (Holcomb & Stewart, 2020; Maes et al., 2022). Apart from contamination prevention, tracking the source of fecal pollution is also important for evaluating any potential risks this pollution may pose to human and animal health. Indeed, water contaminated with animal faeces has been associated with gastrointestinal and other zoonotic diseases (Penakalapati et al., 2017). Furthermore, approximately 60% of human infectious diseases can be spread by animals, while 75% of emerging human diseases appear to have originated from animal sources (Centers for Disease Control & Prevention, 2021). Therefore, contamination of drinking water by animal faeces may also represent an important risk to human health.

Analysing *E. coli* using conventional microbiological techniques generally offers no information about the source of a given strain. To address this, microbial source tracking (MST) techniques can discriminate between sources of fecal contamination in environmental waters (Harwood et al., 2014; Rock et al., 2015). A distinction can be made between library-dependent and library-independent methods: library-dependent methods use a reference library of bacterial strains with a known fecal source to identify the source of unknown strains by comparison, while library-independent methods detect source-informative, host-specific genetic markers (Harwood et al., 2014; Rock et al., 2015; Simpson et al., 2002). Recently, the use of machine learning has also been explored for MST, in combination with the use of *E. coli* as a suitable target for source tracking purposes. Indeed, although this microbe has conventionally been long considered to be a host- and niche-generalist capable of colonizing and transmitting across its various niches (Tenaillon et al., 2010), a growing body of evidence has highlighted a significant degree of niche-specificity within the species (Tiwari et al., 2023; Yu et al., 2021). Machine learning can reliably capture this in the form of host- and niche-specific genetic markers, as shown in the use of a novel logic regression-based method for the identification of host-informative intergenic single nucleotide polymorphisms (SNPs) across the *E. coli* genome (Zhi et al., 2015, 2016a, 2016b). Interestingly, genetic markers identified using logic regression analysis could distinguish between *E. coli* strains from different human and animal host sources with high specificity and sensitivity. They were found to be suitable for source tracking efforts when applied for the source attribution of unknown *E. coli* isolates collected from the Indalsälven river in Northwestern Sweden (Yu et al., 2024).

Since there is no universal method available that can distinguish between sources of fecal contamination, it is helpful to use several methods in tandem. Specifically, combining MST results with Geographic Information System (GIS) tools and environmental DNA (eDNA) monitoring provides a powerful approach for characterizing sources of fecal pollution in aquatic environments (Krolik et al., 2014; Staley et al., 2018). By combining eDNA with MST, potential sources of fecal pollution that are not targeted in the MST analysis can be identified (Staley et al., 2018), which is especially useful for identifying non-point pollution sources. In addition, eDNA can help refine which potential MST targets to look for in an aquatic ecosystem. GIS tools can then complement these other methods by supporting the identification of additional point sources of fecal pollution, or high-risk areas that may contribute pollution to a given watershed (e.g., human/animal densities).

Herein, we present a novel approach to source tracking that integrates *E. coli* enumeration methods with logic regression-based MST and eDNA sampling in a geospatial framework. This approach was tested on an oligotrophic mountain water system in Sweden in an area impacted by tourism and traditional reindeer herding. As such, we sought to unravel the dominant sources of fecal pollution within this oligotrophic mountain area, with a specific focus on pollution from human sources.

## Materials and methods

### Research area and sampling

**Fig. 1:**
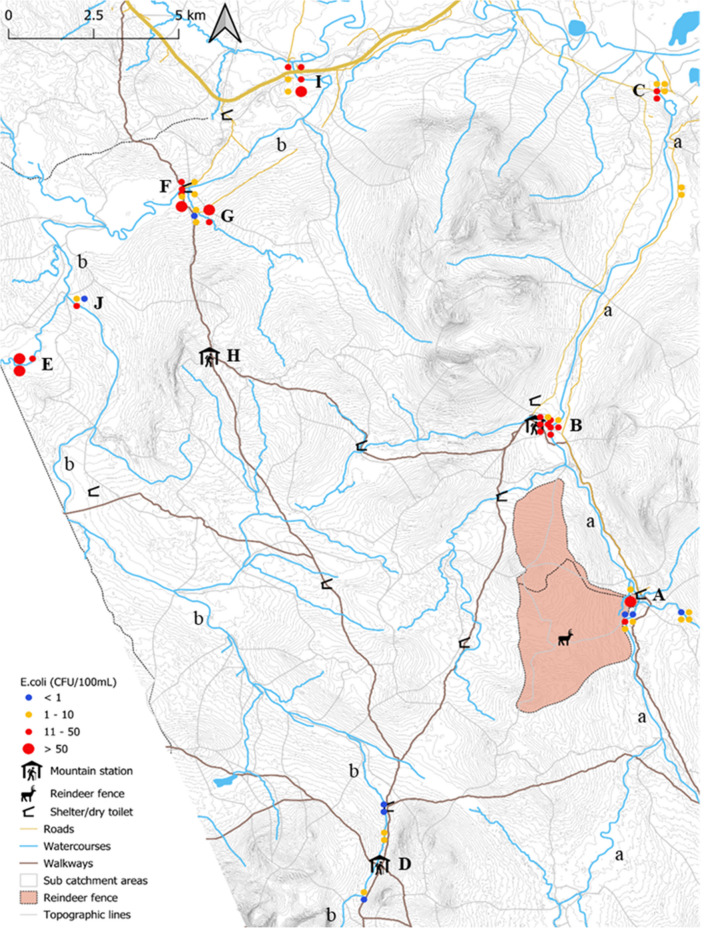
The river basins of Handölan (a) and Enan (b) with an indication of the rivers, tributaries and lakes, highways and roads, walkways, mountain stations, shelters with dry toilet, reindeer fence area and East Norwegian/West Swedish border. Water samples (*n* = 58) are indicated with dots, where the color and size correspond to the *E. coli* level. Each dot represents a water sample for *E. coli* collection and a second water sample for eDNA analysis. Two additional samples were taken 18 km Southeast of Sylarna Mountain Station, upstream and downstream Helags Mountain Station (not shown on map). A: Reindeer fence in Tjallingen, B: Storulvån Mountain Station, C: Handöl village, D: Sylarna Mountain Station, E: Ranglan, F: Sevedholm, G: Tväråbäcken, H: Blåhammaren Mountain Station, I: Enkroken, J: Bridge Enan

This study focused on the river basins of Handölan (454 km^2^) and Enan (325 km^2^) (Fig. 1, a and b), two tributaries in the catchment area of the most upstream part of the Indalsälven River in Jämtland County, Sweden, Europe. Enan has a yearly average water temperature of 3.1 °C and an average river flow rate of 10.7 m^3^ s^−1^, while Handölan´s yearly average water temperature and river flow rate are 2.8 °C and 15.1 m^3^ s^−1^, respectively (SMHI, 2023). These river basins are surrounded by mountain ranges approximately 1700 m above sea level, with the surrounding area containing few roads or permanent settlements (Maes et al., 2022). The main activities impacting the area during the extended summer season include tourism and reindeer herding by the Sami community. An estimated 100,000 tourists and 7000–8000 reindeer (including calves) roam the area each summer. Dogs are also commonly brought to the area by hiking tourists. The dominant wild mammal species commonly found in the summer are moose and beaver, though it can also include otter, hare, red fox, marten, weasel, bear, lynx, wolverine, and smaller rodents.

Sampling spots in the research area were chosen based on previous work exploring the prevalence of *E. coli* (Maes et al., 2022), suspected point sources of fecal contamination, weather conditions, accessibility, and activities impacting the area (e.g., the annual gathering of all female reindeer and their calves for calf marking). A total of 60 water samples were taken for *E. coli* enumeration and MST purposes, alongside 60 additional water samples for eDNA analysis between June 14 and August 18, 2021 (Supplementary data 1). Additionally, 32 beaver and reindeer fecal samples were collected to augment the library of *E. coli* strains used for MST analyses designed for the host identification of the *E. coli* isolates collected from water samples (Yu et al., 2024).

### Collection of metadata in the research area during sampling

The number of tourists in the area around Storulvån Mountain Station on each specific day was estimated using the number of individual crossings at a pedestrian bridge near the station (Data retrieved from the County Administration Board, personal communication, Lansstyrelsen, Tommy Dadell, January 12, 2024).

Water temperature was measured with an analogue thermometer with 0.5 °C precision, with recordings taken following five minutes of submersion. UV radiation was measured using a UV light meter (Center^®^ 532), with the recorded value taken as the average of the minimum and maximum values over one minute. The measured precipitation at the nearest Swedish Meteorological and Hydrological Institute (SMHI) weather station (i.e., Storlien-Storvallen) was used to estimate the precipitation in the whole research area on the corresponding sampling days (SMHI, 2025), and categorized as “high” or “low” after comparison with the average monthly precipitation levels (i.e., 6.4 mm, 2.7 mm, and 6.4 mm for June, July, and August, 2021, respectively) at the same weather station.

The modelled water flow rate data at the corresponding sampling locations and days were retrieved from the SMHI website (SMHI, 2023). Calculations for the sub-catchment areas were performed with the hydrological model S-HYPE. SMHI validated the model using the total water flow data measured at several stations along the river, thereby representing the model-calculated water flow at the outlet of the chosen sub-catchment area, including contributions from all possible sub-catchment areas upstream of the selected location. An assessment of the river flow rate as high or low was made after comparison with the average modelled monthly flow rate.

All additional information related to the environmental conditions (i.e., tourism, water temperature, UV radiation, precipitation, and river flow rate) at each sampling location is included in the supplementary material (Supplementary data 1).

### Microbial source tracking of *E. coli* isolates

#### Collection of *E. coli* isolates and DNA extraction

Fecal samples were collected from a variety of animal hosts for *E. coli* enumeration and isolation (i.e., for MST) purposes. One gram of each fecal sample was diluted in 100 mL of Peptone water (Oxoid, LP0037), serially diluted, and then passed through a 0.45 µm filter (ReliaDisc™, Ahlstrom-Munksjö, 760,245): The filters were transferred to Membrane Faecal Coliform Agar (mFC, Difco™ mFC agar, BD Biosciences, 267,720) with 0.01% Rosolic acid (Difco™ Rosolic acid, BD Biosciences, 232,281), and incubated at 44 ± 0.5 °C for 22 ± 2 h. Morphologically distinct blue colonies on the mFC plates, representing potential *E. coli*, were inoculated in Lactose Tryptone Lauryl Sulphate Broth (LTLSB, Oxoid, CM0921) supplemented with 4-methylumbelliferyl-β-D-glucuronide (MUG supplement, Oxoid, BR0071E) and incubated for 21 ± 3 h at 44 ± 0.5 °C for confirmation. Tubes that fluoresced under UV light were confirmed to be *E. coli* strains, and then stored at −18 °C in Brain Heart Infusion Broth (BHI, Oxoid, CM1135) with 20% glycerol (Apl, 33868), according to their original morphological appearance (i.e., to reduce clonal representation in the library). In this way, a total of 5 reference isolates from beaver and 52 from reindeer were collected.

River water samples for *E. coli* enumeration and isolation, were collected in sterilized plastic 500 mL bottles. All samples were collected by hand as far away from the shore as possible by completely submerging the bottles under the water surface with the opening facing upstream to avoid contamination from the sampler. After sampling, the water bottles were stored at 0–7 °C in an electric cooler (storage time given in Supplementary data 1). *E. coli* enumeration was performed in the lab by passing 10 mL and 100 mL of each water sample through a 0.45 µm filter. Further processing and isolate collection were performed as described earlier. The limit of quantification for the enumeration of *E. coli* was 1 CFU/100 mL.

To ensure no contamination had occurred during handling, all stored *E. coli* isolates were inoculated from their BHI glycerol stocks on mFC and incubated at 44 ± 0.5 °C for 22 ± 2 h. Only isolates that showed a typical blue *E. coli* morphology were chosen for DNA extraction using DNeasy^®^ Blood & Tissue Kits (Qiagen, 69,506) according to the manufacturer´s instructions. The quality and quantity of the purified DNA for each isolate were checked using a Nanodrop 2000 (Thermo Scientific), and DNA extracts were stored at −20 °C.

#### Amplification, sequencing, and analysis of intergenic regions

Two intergenic regions (ITGRs) that were previously shown to be host-informative (Yu et al., 2024; Zhi et al., 2015, 2016a, 2016b) were selected for logic regression analysis, including: 1) the *csgBAC-csgDEFG* region, encoding regulators of the synthesis of curli fimbriae, and 2) the *asnS-ompF* locus encoding a regulator of an outer membrane protein related to low osmolality conditions. These two regions were amplified separately using the same PCR reaction mixture and cycling conditions. Each PCR reaction contained 10 µL of genomic *E. coli* DNA, 10 µL KAPA2G Buffer A 5× (Merck, KK5005), 10 µL KAPA Enhancer1 5× (Merck, KK5005), 2 µL MgCl_2_ 25 mM (Merck, KK5005), 1 µL of each primer 25 µM (Table 1, Sigma-Aldrich), 1 µL dNTPs 10 mM (Merck, KK5005), 0.4 µL KAPA2G Robust DNA Polymerase 5 U/µL (Merck, KK5005), and 14.6 µL of sterile distilled water.

**Table 1:** PCR primers

Target	Primer	Sequence (5´ → 3´)	Reference
csgBAC-csgDEFG	csgD-1	GGACTTCATTAAACATGATG	Zhi et al., 2015
	csgD-2	TGTTTTTCATGCTGTCAC	Zhi et al., 2015
asnS-ompF	ompF-F	TACGTGATGTGATTCCGTTC	Zhi et al., 2015
	ompF-R	TGTTATAGATTTCTGCAGCG	Zhi et al., 2015
12S rRNA gene	F1	ACTGGGATTAGATACCCC	Kelly et al., 2014
	R1	TAGAACAGGCTCCTCTAG	Kelly et al., 2014

PCR amplifications were performed on an automated thermal cycler (LifeTouch) with an initial denaturation (4 min at 95 °C) followed by 33 cycles of denaturation (30 s at 95 °C), annealing (30 s at 58 °C) and extension (1 min at 72 °C) and a final extension (7 min at 72 °C). PCR products were subject to gel electrophoresis (CBS Scientific, SGE01402 and Consort, E863) in a 1% agarose gel (VWR, 438792U), TBE buffer 1× (diluted from TBE buffer 10×, Invitrogen, 15,581–044) and GelRed^®^ Nucleic Acid Gel Stain 1× (Biotium, 37–41,001) and visualized following exposure to UV light. All PCR products that showed one band of the desired size were excised, sequenced bidirectionally using Sanger sequencing by Macrogen Inc. (Amsterdam), and then aligned with Clustal Omega (Sievers et al., 2011). The aligned sequences for each ITGR were then manually edited to trim the 3’ and 5’ ends to remove missing data, and then concatenated into a single sequence for each *E. coli* isolate for logic regression analysis using the R code as described in Yu et al. (2024).

All concatenated sequences were added to an existing library of ITGR sequences from *E. coli* strains (available in Supplementary data 2) collected from various hosts (Zhi et al., 2016a, 2016b). Logic regression was then used to analyse the sequence variation contained within these aligned ITGR sequences to identify host-informative SNP markers that can then be used for MST purposes. The ITGR sequences of the river water *E. coli* isolates (i.e., from unknown host sources) were then compared to each host-specific logic model to classify them according to their potential original host source, as previously outlined by Yu et al. (2024).

### eDNA analysis

#### Collection of eDNA samples and extraction

eDNA samples were collected from downstream to upstream locations to avoid introducing incidental contamination. All sampling supplies were first cleaned with bleach (10% NaClO) and then rinsed with clean water before sampling. Gloves and mouth masks were used to further prevent contamination of the samples. For eDNA sampling, kits provided by AquaBiota Water Research were used. In cases where the water surface could be easily reached, samples were taken directly from the river. If this were not the case, five subsamples of the river were collected using buckets, and 1 L of each subsample was pooled together. A minimum of 1500 mL and maximum of 3000 mL (Supplementary data 1) for water samples (depending on the number of total particles in the water) and 1500 mL for field negatives were filtered over enclosed double filter, 5 μm glass fibre/0.8 μm polyether sulfone membranes (NatureMetrics Ltd, United Kingdom). Air was then pushed through the filter three times to remove any remaining water. All samples were fixed with 96% molecular grade ethanol, and the ends of the filter were closed with locks and parafilm to minimize the risk of leakage. All filters were collected in clean plastic bags, stored at 7 °C, and then sent to the laboratory (MoRe Research AB) for DNA extractions. Filters were stored at 7 °C until DNA extraction on all samples could be performed.

DNeasy^®^ Blood & Tissue Kits (Qiagen, 69,506) were used for all eDNA extractions. The supplier’s protocol was used with optimizations by Spens et al. (2017) and further modified by Kačergytė et al. (2021) for eDNA analysis. These modifications involved pooling the lysate, following the overnight lysis of the filter capsule DNA and the ethanol pellet DNA into one sample. The extracted samples were sent to a commercial laboratory (Nature Metrics Ltd, United Kingdom) for further analysis.

#### Metabarcoding of eDNA samples

All extracted eDNA samples were meta-barcoded following the “DNA survey – Vertebrates pipeline” from Nature Metrics Ltd. Purified DNA was used for PCR analysis to amplify a hypervariable region of the 12S rRNA gene, using primers described by Kelly et al. (2014) (Table 1). The analysis included 12 replicate PCRs per sample and included both negative and positive control samples. PCR replicates were pooled and purified, and sequencing adapters were added. Amplicons were purified and subsequently quantified using a Qubit broad-range kit according to the manufacturer´s protocol. Sequencing was performed using an Illumina MiSeq V3 kit at 10.5 pM with a 20% PiX spike in. The resulting sequence data were processed using a custom bioinformatics pipeline for quality filtering, operational taxonomic unit (OTU) clustering, and taxonomic assignment. The OTU table was filtered to remove low-abundance OTUs from each sample (< 0.025% or < 10 reads, whichever was the greatest threshold for the sample). After this, taxonomic information was added by sequence similarity searches against the NCBI *nt* reference database (Genbank). The presented species level identification reflected the top hit based on species identity with a minimum of 99% similarity. When equally good matches with multiple species were observed, public records from the Global Biodiversity Information Facility were used to assess the match with the highest likelihood based on each species´ presence in Sweden.

## Results

### Microbial enumeration in water samples across the research area

*E. coli* enumeration varied between locations and sampling dates throughout the research area (Fig. 1) from < 1 CFU/100 mL to 210 CFU/100 mL. The high frequency of yellow and red dots indicates that the water is generally not suited as a direct source of drinking water (without pretreatment such as boiling or filtering). Samples taken in the river basin of Enan contained significantly higher *E. coli* levels than the river basin of Handölan (p = 0.021).

The highest *E. coli* prevalence in Handölan was at the *tributary within the reindeer fence* and around the *Storulvån mountain station* (Fig. 1, A and B). It should be noted that high *E. coli* counts in *the tributary within the reindeer fence* were only observed on one occasion when approximately 5000 female reindeer and calves were gathered for two days for calf marking. *E. coli* originating mainly from reindeer was therefore expected in the fenced tributary on that occasion. In contrast, low *E. coli* counts were observed at this site during the month before the reindeer gathering. The area further downstream around the *Storulvån mountain station* was expected to reflect contamination from human and human-associated animal sources (i.e., dogs) as well as from wild animals and reindeer.

The corresponding hotspots for Enan were at the tributaries *Ranglan* and *Tväråbäcken* (Fig. 1, E and G) and at the two downstream locations in the main river at *Sevedholm* and *Enkroken* (Fig. 1, F and I). The tributary *Ranglan* comes from a remote area in Norway with little or no human infrastructure. The tributary and main river are located within a large wetland area with many observed beaver dens, thus, the dominant source of *E. coli* in this tributary was thought to come from wild animals, including beaver and reindeer. *Sevedholm* is a resting area for tourists and a popular fishing and bathing site. *Enkroken* is a downstream reference point where the Indalsälven Water Conservation Association has monitored water quality six times per year since 1993 (IWCA, 2024). The second tributary (*Tväråbäcken*) comes from the area around the *Blåhammaren mountain station* (Fig. 1, H), where there have been frequent problems with the sewage treatment due to inadequate conditions for proper infiltration given the rocky terrain and very thin soil. Consequently, human sources of *E. coli* were expected to dominate at these locations, although contributions from wildlife and reindeer could not be excluded. These sampling sites are discussed in more detail in the sections below.

### Microbial source tracking of *E. coli* isolates

One hundred and ninety-five *E. coli* strains were collected from water samples from the research area. For 113 of those strains, the 2 target ITGRs were successfully amplified, concatenated, and analysed by logic regression analysis. With a sufficient level of consensus (i.e., ≥ 80% across all classification iterations, achieving congruency as previously described by Yu et al. (2024)), 16, 13, and 19 of the *E. coli* isolates could be classified as originating from human, reindeer, and beaver, respectively. Two isolates showed an indistinguishable match with all three models and were left unclassified, while the remaining 63 *E. coli* isolates were inconsistently classified and were also consequently reported as unclassified.

**Fig. 2:**
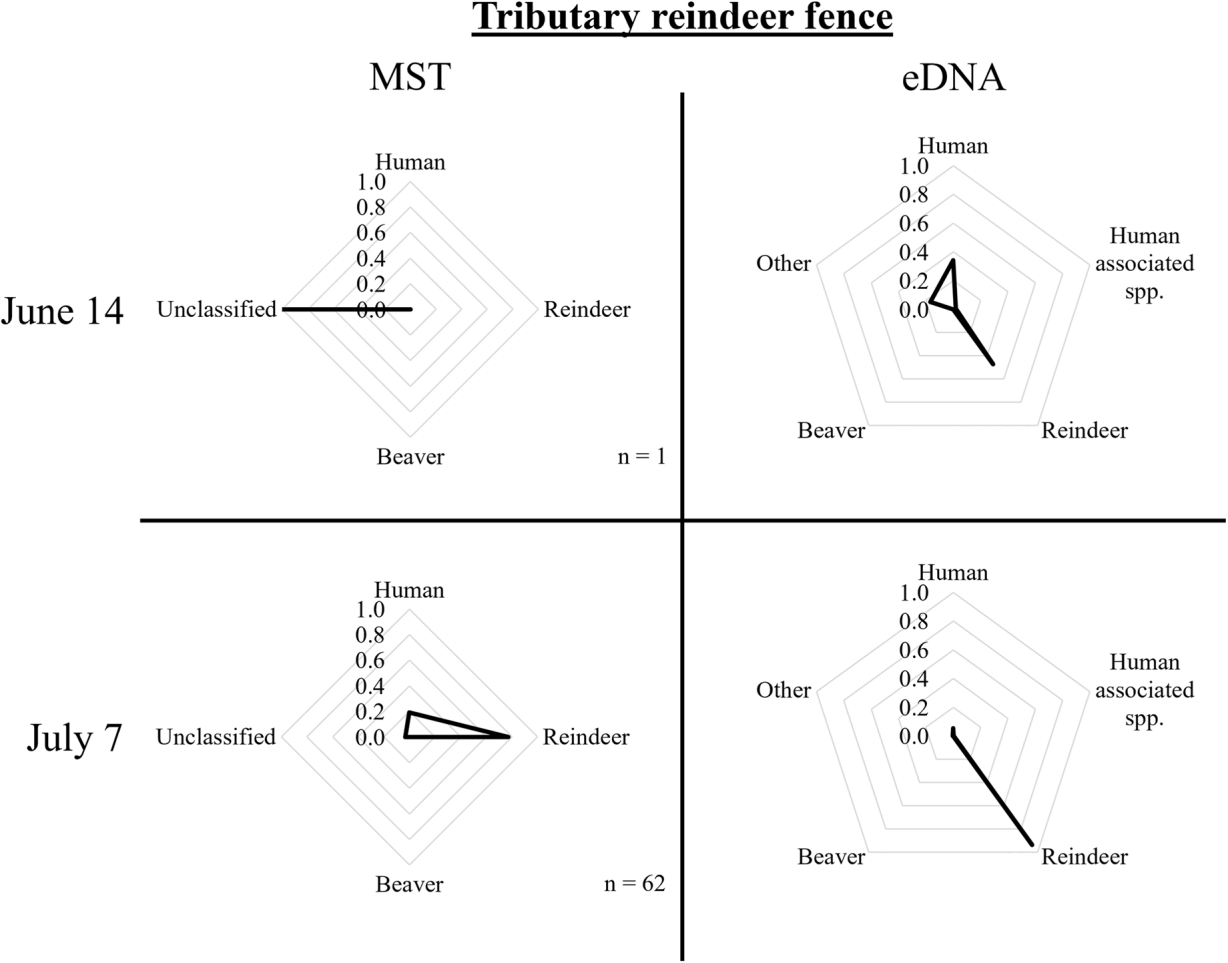
MST and eDNA results from samples taken in the tributary originating from the reindeer fence on June 14 and July 7, 2021. The number of *E. coli* strains per sampling occasion applied in the logic regression models for classification is indicated by n

**Fig. 3:**
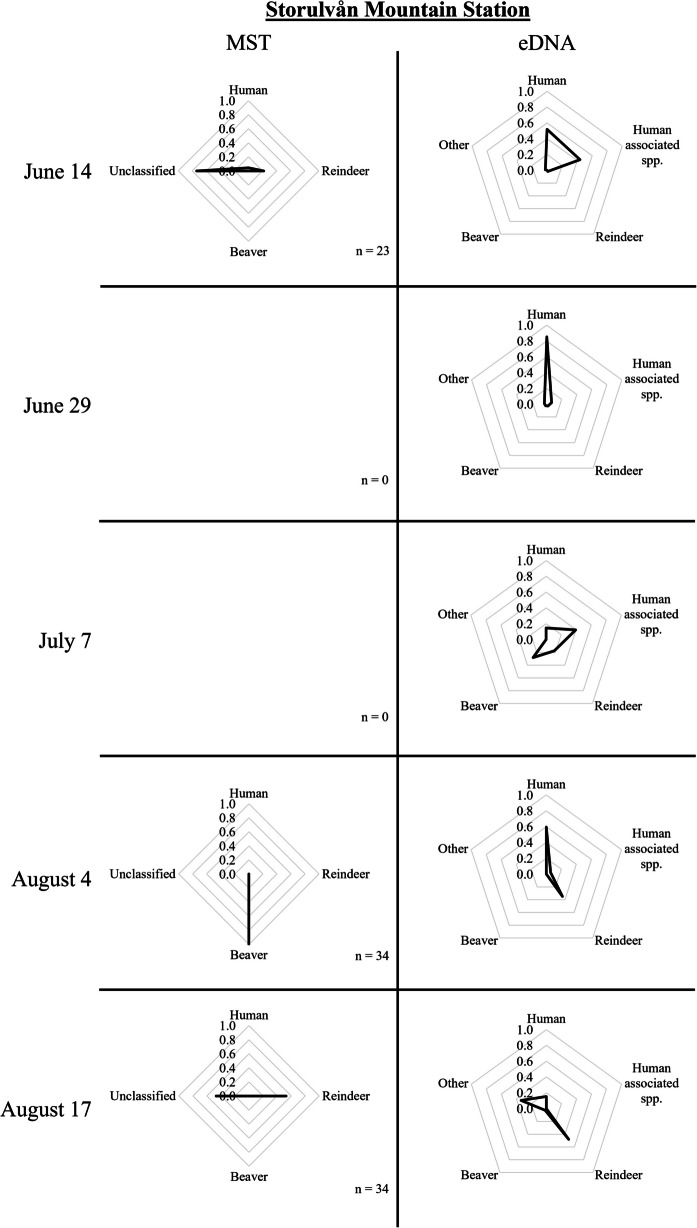
MST and eDNA results from samples taken downstream Storulvån Mountain station on June 14, June 29, July 7, August 4, and August 17, 2021. The number of *E. coli* strains per sampling occasion applied in the logic regression models for classification is indicated by n

In the catchment area of Handölan, *E. coli* collected in the *tributary within the reindeer fence* (Fig. 1, A) were mostly classified as originating from reindeer, though there was also a significant proportion of unclassified strains and a small proportion of human strains (Fig. 2). Interestingly, the proportion of strains classified as originating from reindeer increased drastically from when reindeer were roaming freely in the area (June 14/15) to when they were gathered in the fenced area for calf marking (July 7/8). The proportion of human strains also increased, in a less pronounced way, between the two distinct situations. Further downstream, near the *Storulvån Mountain Station* (Fig. 1, B), most *E. coli* strains were classified as beaver- or reindeer-derived (Fig. 3); however, at *Handöl village* (Fig. 1, C), which collects all water in the Handölan catchment area, most *E. coli* strains were left unclassified, with a small portion originating from beaver and reindeer.

**Fig. 4:**
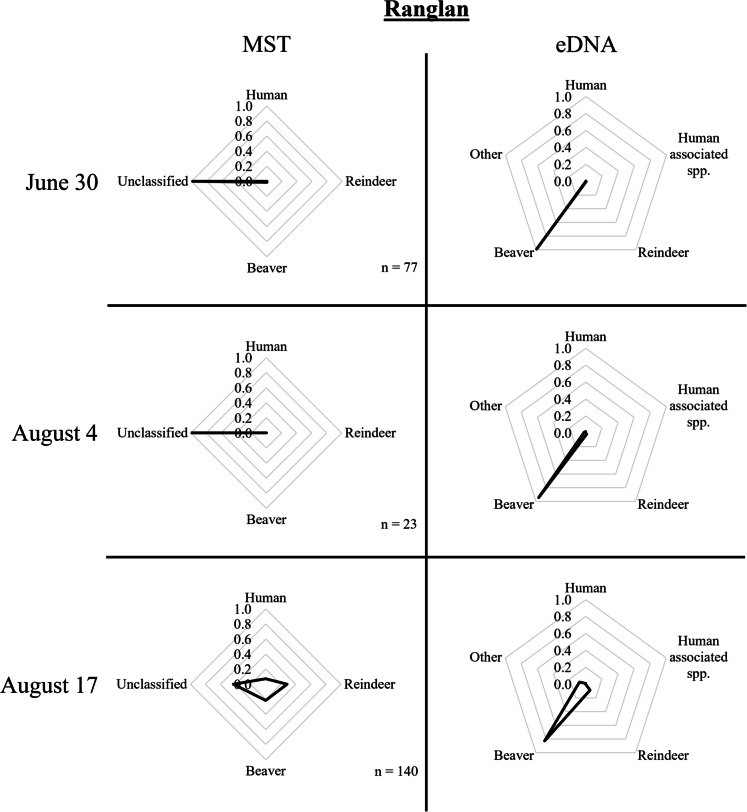
MST and eDNA results from samples taken in Ranglan on June 30, August 4, and August 17, 2021. The number of *E. coli* strains per sampling occasion applied in the logic regression models for classification is indicated by n

**Fig. 5:**
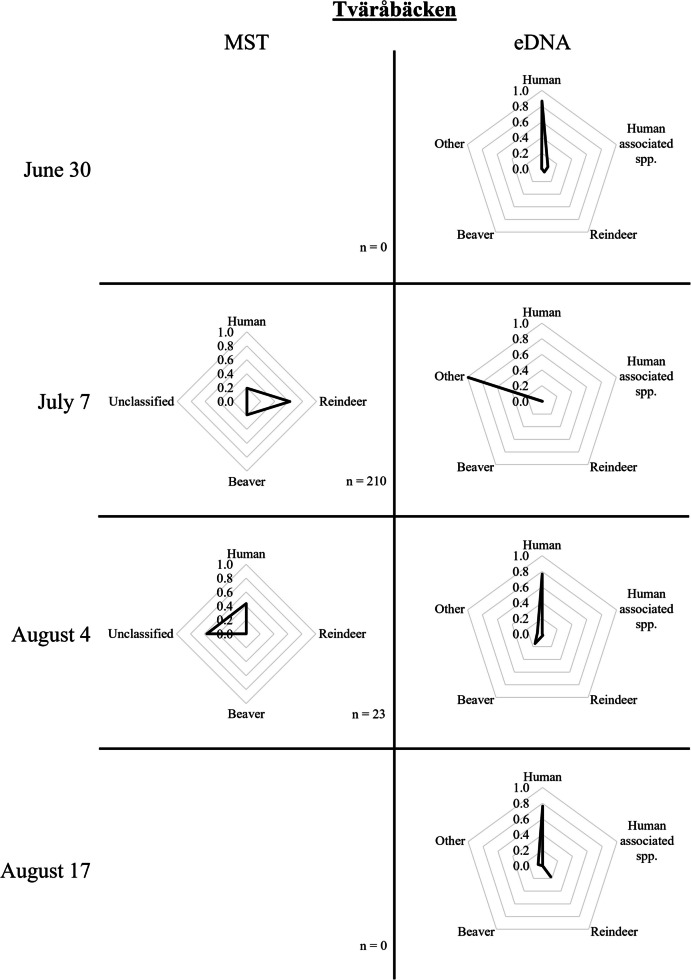
MST and eDNA results from samples taken in Tväråbäcken on June 30, July 7, August 4, and August 17, 2021. The number of *E. coli* strains per sampling occasion applied in the logic regression models for classification is indicated by n

**Fig. 6:**
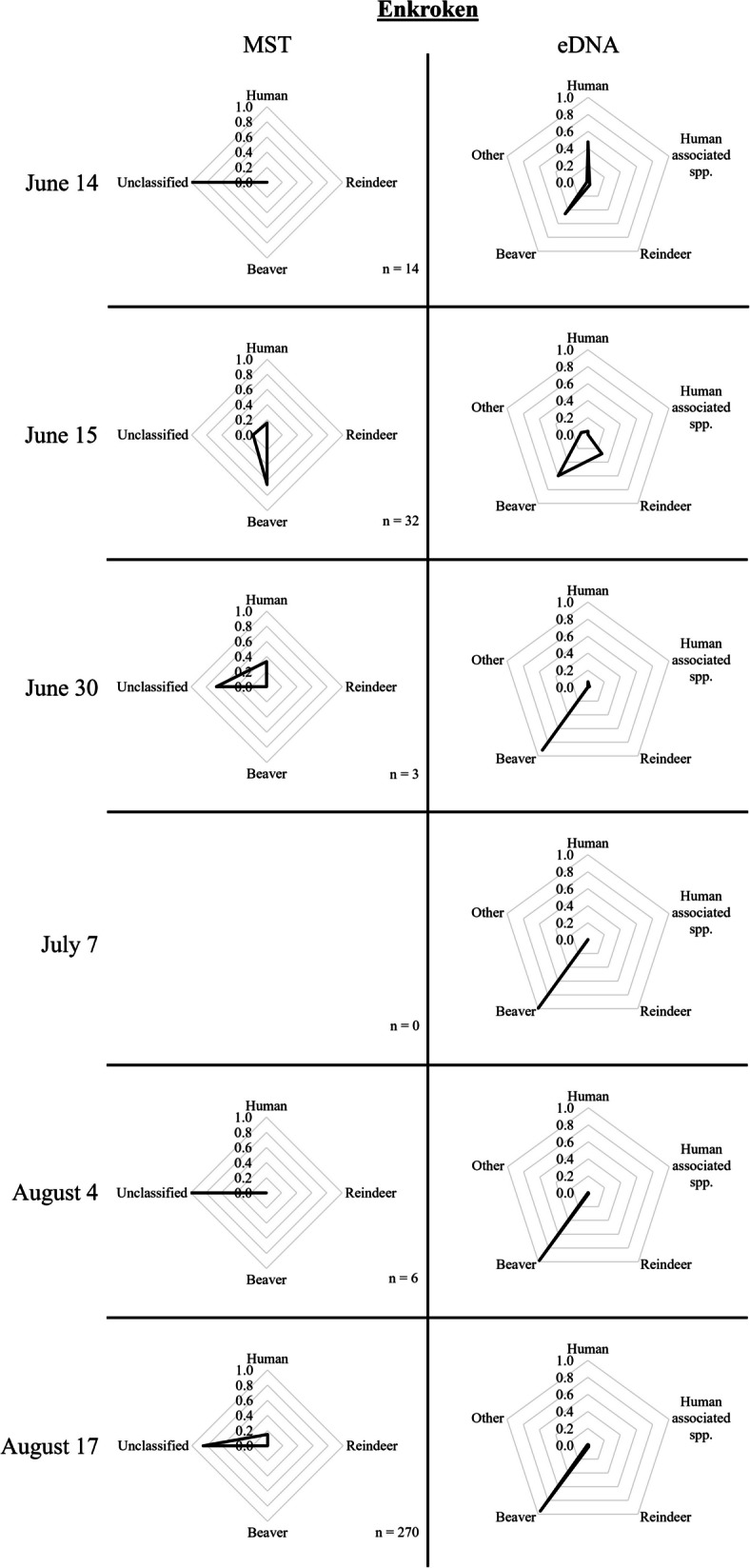
MST and eDNA results from samples taken at Enkroken on June 14, June 15, June 30, July 7, August 4, and August 17, 2021. The number of *E. coli* strains per sampling occasion applied in the logic regression models for classification is indicated by n

Few to no *E. coli* were recovered in the area around the *Sylarna Mountain Station* (Fig. 1, D), the most upstream sampling location in the Enan tributary, and consequently, limited MST data were generated for this area. The tributary to Enan, *Ranglan* (Fig. 1, E), contained many unclassified strains but also strains originating from reindeer, beaver, and human in increasing prevalence (Fig. 4). Further downstream, around the rest area *Sevedholm* (Fig. 1, F), most strains were again unclassified, but an increase in the contribution of beaver strains was observed. On one specific sampling occasion (July 7/8) when *E. coli* numbers were particularly high (31 CFU/100 mL), the proportion of human strains was much higher than at other times. At this location, there is also an incoming tributary (i.e., *Tväråbäcken*, Fig. 1, G) collecting water from the area around the *Blåhammaren Mountain Station* (Fig. 1, H). This creek contained more reindeer and human *E. coli* strains, with fewer strains remaining unclassified (Fig. 5). At *Enkroken* (Fig. 1, I) most strains remained unclassified, though some strains could be classified as originating from human and beaver sources (Fig. 6).

Interestingly, samples taken at locations representing a large catchment area (> 200 km^2^) contained significantly larger proportions (p = 0.019) of *E. coli* strains that remained unclassified (64%) following logic regression analysis compared to locations that represent a small catchment area (< 50 km^2^, 23%).

### eDNA analysis

High-quality vertebrate sequence data were obtained for all the eDNA samples, detecting 12 different mammal species: beaver, dog, human, mink, moose, otter, pig, reindeer, shrew, vole, whitetail deer and wolverine. The results are presented as the proportion per species represented in a sample, where the focus was on human, dog and pig (collectively reported as “human-associated species”), as well as reindeer and beaver sources. The other mammalian species that contributed to the DNA content of a sample were grouped and reported as “other”.

The samples taken from the *tributary within the reindeer fence* (Fig. 1, A) mostly contained reindeer and human DNA (Fig. 2). The samples upstream of the reindeer fence additionally contained a significant proportion of beaver DNA. In the *tributary within the reindeer fence*, there was an increase in the proportion of reindeer DNA (and a drop in human DNA) when the reindeer were gathered for calf marking within the fenced area (July 7/8) compared to when the reindeer were roaming freely in the whole research area (June 14/15). Samples taken near the *Storulvån Mountain Station* (Fig. 1, B) were mostly dominated by human or human-associated species DNA (Fig. 3). Only the samples taken in August contained less than 50% of human DNA, supplemented by reindeer DNA or DNA from other mammals. At *Handöl village* (Fig. 1, C), the eDNA samples contained beaver DNA and, to a lesser extent, human, reindeer, and other DNA.

For samples taken around the *Sylarna Mountain Station* (Fig. 1, D), eDNA results showed a significant contribution from human DNA. In August, the reindeer DNA proportion was higher than the samples taken in July. Around 22 km downstream of this mountain station, eDNA samples taken at a footbridge (i.e., *Enan bridge*, Fig. 1, J) were still dominated by human DNA but also contained significant proportions of reindeer and other DNA. In *Ranglan* (Fig. 1, E) and the rest area of *Sevedholm* (Fig. 1, F), located further downstream, all samples were dominated by beaver DNA (Fig. 4). *Tväråbäcken* (Fig. 1, G), the creek collecting water from the area around the *Blåhammaren Mountain Station,* mostly contained human DNA, however, at one sampling occasion (July 7/8), the sample in *Tväråbäcken* consisted almost completely of moose DNA (Fig. 5). The reference point for Enan, i.e., *Enkroken* (Fig. 1, I), contained mostly beaver DNA, with only samples that were taken on June 14/15 containing a significant portion of human and reindeer DNA (Fig. 6).

The number of species other than human, human-associated species, reindeer and beaver were similar between large (> 200 km^2^) and small (< 50 km^2^) catchment areas (p = 0.421) but the proportion of DNA contributed by these other mammal species varied significantly (p = 0.048), with an average of 6% for large catchment areas and 14% for small catchment areas.

### eDNA and MST results from locations of specific interest

Data from specific locations of interest are presented in more depth in the following section. The reason for this is that they: 1) represent a location with well-known activities in relatively small catchment areas (i.e., the *tributary within the reindeer fence* for reindeer activity, *Ranglan* for beaver activity, and *Tväråbäcken* for human activity) or 2) represent a large catchment area including many potential contamination sources (i.e., *Storulvån Mountain Station* and *Enkroken*).

Water samples from the *tributary within the reindeer fence* were taken on two occasions (Fig. 2), from a relatively small catchment area of about 5 km^2^ with very little to no tourist activity and few reindeer present over the summer, except for the very short time they were gathered for calf marking. *E. coli* counts increased from 1 to 62 CFU/100 mL during increased reindeer and human activity in the area on July 7 compared to June 14. MST of the only collected *E. coli* isolate on June 14 was left unclassified, while 77.4% and 19.4% of the collected strains on July 7 were classified as originating from reindeer or human, respectively. Both eDNA samples were dominated by reindeer and human or human-associated species, but the proportion of reindeer DNA increased on July 7 when more reindeer were present in the area.

Further downstream in Handölan, samples were taken below the *Storulvån Mountain Station* (Fig. 1, B) with a catchment area of approximately 420 km^2^. The estimated number of tourists in the area around the station over the extended summer season (May 1 until October 31, 2021) was approximately 43,000, during which *E. coli* numbers varied between 4 and 35 CFU/100 mL. *E. coli* strains collected on June 14, August 4, and August 17 were used in the MST models for classification. On June 14, one strain appeared to originate from a human host, and five strains originated from reindeer hosts, but besides these strains, most were left unclassified. Strains collected on August 4 were all classified as originating from beaver, while the sample taken on August 17 contained a mix of reindeer and unclassified strains. Most eDNA samples taken at this location were dominated by DNA from human or human-associated species. Samples taken in July and August had a larger fraction of reindeer eDNA compared to the samples taken in June. The sample taken on July 7 also contained a significant proportion of beaver DNA (28%), and the sample taken on August 17 was the only sample containing a significant proportion of eDNA coming from other mammals (Fig. 3).

One of the tributaries to Enan, *Ranglan* (Fig. 1, E), was sampled on three different occasions. This sampling point collects water from a catchment area of approximately 44 km^2^, which is mainly situated in a remote Norwegian territory. This area is believed to be affected mostly by wildlife (such as beaver and reindeer), with little to no human activity. *E. coli* counts were always high, ranging from 24 to 98 CFU/100 mL. Most of the *E. coli* strains collected at this location remained unclassified, though strains collected on August 17 could be attributed to reindeer (29%), beaver (21%), and human (7%) sources. Despite this, all eDNA samples showed a clear dominance of beaver (Fig. 4).

Further downstream, the *Tväråbäcken* tributary consisted of a catchment area of 16 km^2^, which included the area around the *Blåhammaren Mountain Station*. It should be noted in this context that the sewage treatment plant at Blåhammaren Mountain Station has had recurring problems leading to the release of untreated sewage to the recipient area on occasions. Apart from this, the main sources of contamination are expected to be from hikers, reindeer, and other wildlife in the area. *E. coli* counts varied widely from 2 to 210 CFU/100 mL. At this location, *E. coli* strains in the sample taken on July 7 (containing 210 CFU/100 mL *E. coli*) mainly originated from reindeer (62%), human (19%), and beaver (19%). In contrast, the sample taken on August 4 contained strains that were mainly unclassified (57%) or human-derived (43%). Most eDNA samples taken in *Tväråbäcken* were dominated by human DNA, though in some samples this was complemented by DNA from reindeer, beaver, human-associated species, and other mammals. The only exception was the sample taken on July 7, in which 99% of the eDNA belonged to other mammals (i.e., moose, Fig. 5).

At the reference point of Enan (i.e., *Enkroken*), water quality monitoring has been performed since 1993. This location receives water from a large catchment area of ~ 325 km^2^, including two mountain stations, *Blåhammaren* and *Sylarna,* and includes many kilometers of hiking trails and shelters as well as wildlife and reindeer herding in the wetlands of the lower lying parts of the Enan valley. During this study from June until August, 2021, *E. coli* counts fluctuated from 4 to 155 CFU/100 mL. Interestingly, the number of *E. coli* increased considerably (from 15 to 33 CFU/100 mL) on two consecutive days (June 14/15) with heavy rainfall. Using MST, 79% of all strains collected at this location were left unclassified, while the remaining 21% could be attributed to human (14%) and beaver (7%) sources. Interestingly, the water sample taken on June 14 contained only unclassified strains, while the sample taken following heavy rainfall contained strains that were classified as beaver and human origin. All eDNA samples were dominated by beaver DNA, though samples taken on June 14/15 contained a slightly more diverse eDNA pattern. The sample taken on June 14 contained mostly DNA originating from human and human-associated species (50%), followed by beaver (46%) and reindeer (4%), which shifted to predominantly beaver (60%), followed by reindeer (28%), other mammals (8%), and human (4%) on the day after heavy rainfall (Fig. 6).

## Discussion

### General findings from microbial enumeration, MST, and eDNA analysis

The *E. coli* levels found in this study (< 1 CFU/100 mL to 210 CFU/100 mL) may seem low compared to levels that are commonly found in many rivers worldwide. Against this background, these clear and nutrient-poor aquatic ecosystems appear unique from an international perspective (Jonsson & Agerberg, 2015; Maes et al., 2022) and evidence of fecal pollution should therefore be taken seriously. Measurements carried out at *Enkroken* by the IWCA (2024) showed that at the onset of the monitoring program in 1993, the water quality frequently met national standards for drinking water without purification, but also that the situation has gradually worsened since then. The results from this study highlight the fact that the water quality is impacted by fecal contamination that frequently makes it unsuitable as a direct source of drinking water.

Similar to our study conducted in 2020 (Maes et al., 2022), *E. coli* counts appeared to show a complex pattern across the research area over the summer of 2021. At all but two of the investigated locations where fresh fecal contamination was established based on *E. coli* counts, at least part of the contamination could be allocated to a host source based on MST results. At specific locations, we were able to demonstrate the contribution of human, reindeer, and/or beaver fecal pollution to the mountain rivers of the research area. Despite this, many of the collected *E. coli* strains could not be classified by logic regression and were not allocated to a host source. The large fraction of unclassified *E. coli* strains raises questions about the origin of these isolates and their supposed host source(s). As discussed in a previous paper (Yu et al., 2024), there are several possible explanations for the high proportion of unclassified strains in these samples. First, there may be areas of improvement (i.e., increasing strain representation, using other ITGRs, etc.) for the MST workflow to increase the classification power of the logic regression algorithm. Secondly, various other mammal species (i.e., moose, vole, lemmings, etc.), that could potentially introduce fecal contamination to the sampling sites, were not represented in the MST classification scheme and *E. coli* isolates derived from these alternative host sources could comprise a proportion of the environmental isolates that were left unclassified. Thirdly, some of the unclassified isolates could alternatively have belonged to naturalized *E. coli* populations that have become adapted to the natural environment as a primary niche. Considering that distinct naturalized populations have been described to reside in river water, sediments and even wastewater (Jang et al., 2011, 2015, 2017; Zhi et al., 2016a), some of the river water isolates that were left unclassified in this study could instead represent naturalized strains not captured by a classification workflow focused on host source attribution. Finally, the MST approach used in this study focused on the identification of host specialists in a population (i.e., those exclusive to a single animal host), thereby leaving *E. coli* generalists (i.e., multiple hosts) unclassified in the analysis.

Interestingly, there was a distinct relationship between the fraction of unclassified *E. coli* strains and the size of the catchment area, where samples taken within small catchment areas often had a low fraction of unclassified strains, whereas samples taken within large catchment areas often had a large fraction of unclassified strains. However, the eDNA results did not suggest a significantly greater contribution from mammals other than humans, reindeer, and beaver in the large catchment areas, reducing the likelihood that the large proportion of unclassified strain in these areas originated from mammal sources that were not targeted in the MST analysis. As the *E. coli* inactivation rate is expected to be low in these cold and oligotrophic rivers (Blaustein et al., 2013; Jonsson & Agerberg, 2015), transport distances from the contributing source to the sampling points may be considerable. This could affect the viability of *E. coli* originating from human/animal host populations in the distal reaches of the watershed without affecting the stability of the eDNA signatures.

On average, 90% of the eDNA from the research area could be attributed to human, human-associated species (i.e., dog and pig), reindeer, and beaver sources. It was only on two occasions that more than 50% of the DNA in a sample originated from other mammals. This confirms the results from exploratory samples taken in the research area in 2018 and 2020 (unpublished reports from Naturhistoriska Riksmuseet 4.1–147-2019 and Nature Metrics 101,807, respectively), showing that the most dominant sources of mammal DNA contributing to this research area originated from human, beaver, and reindeer populations. For locations representing a small catchment area, we can deduce which mammals likely contribute fecal contamination to the surrounding water based on knowledge about the area and ongoing activities. For these types of locations, there is generally a good connection between the MST and eDNA results. For example, it was predicted that during reindeer calf marking, contamination from human and reindeer sources would increase in the *tributary within the reindeer fence* compared to the rest of the summer when reindeer were scattered across the whole research area and when there is little human activity inside the fenced area, which was confirmed by both MST and eDNA results. Another location representing a small catchment area with known activities (i.e., concentrated human activity around *Blåhammaren Mountain Station* and wildlife in the lower part of the Enan valley) is *Tväråbäcken*. At this location, human activity was mostly confirmed by the eDNA results. For samples representing large catchment areas, it is much more difficult to predict the predominant host sources of fecal pollution, since the area includes many more potential contributing sources that can impact the surrounding waters. For these locations, MST and eDNA results usually did not correlate notably, as a large proportion of *E. coli* strains collected in these areas were left unclassified by MST. For locations where there is little information about possible contamination sources (e.g., *Ranglan)*, eDNA can provide useful insight since this method is not restricted to the targeted animal sources as in the MST approach. Accordingly, MST data alone did not show the dominant presence of beavers in this area, likely due to the large proportion of unclassified strains, even though eDNA results identified beavers as the predominant host source impacting the surrounding waters in this area.

In this study, we have shown that wildlife, including beavers and domestic reindeer, are important sources of fecal pollution in the research area. This contamination can be considered the baseline pollution level in such mountain rivers, since wildlife is naturally present, and the Sami community has been practicing traditional reindeer herding for centuries. On top of this basic/natural pollution level, fecal contamination in the mountain rivers from human sources appears to be significant and mostly a result of increasing tourism in the area. Our findings are in accordance with many studies included in the review by Paruch and Paruch (2022), in which humans were often identified as the main source of fecal pollution in freshwater river systems. Exposure to this pollution source can increase risk due to exposure to potentially harmful pathogens (Ågerstrand et al., 2023; Bong et al., 2022), which can in turn impact the Indigenous Sami community and their way of life, as it is highly dependent on a clean environment.

### Evaluation of the used approach for source tracking of fecal pollution in oligotrophic mountain waters

In this study, a combination of three methods (i.e., microbial enumeration, MST, and eDNA analysis) was used to track sources of fecal contamination in oligotrophic mountain rivers. Microbial enumeration, specifically *E. coli* colony counts, is traditionally used for monitoring the quality of water intended for human consumption, however, this method alone is not suitable for tracking potential contamination sources (Paruch & Paruch, 2022). Rather, knowing the sources of fecal pollution allows for mitigation measures to reduce or even prevent pollution, and will ultimately lead to a better estimation of the possible impacts of fecal contamination from a “One Health” perspective (Centers for Disease Control & Prevention, 2024).

Therefore, microbial enumeration was complemented with a logic regression-based MST approach to determine the potential source of *E. coli* isolates collected in this study. This method was successful in attributing a portion of the detected *E. coli* in the research area to their respective host origins; however, the MST analysis was mostly restricted by the preselected target species that were used to develop the reference library. Species not represented in the reference library were thus unable to be attributed to the *E. coli* strains collected from river samples. In this study, the chosen host sources were based on the main activities in the area (i.e., tourism, reindeer husbandry, and wildlife) and the results of exploratory eDNA analyses showing the dominant presence of human, beaver, and reindeer DNA. Ideally, the selection of host sources should correlate strongly with the water sources and the surrounding area in question (Harwood & Stoeckel, 2011). However, due to the limited number of isolates collected, we supplemented our library with human *E. coli* sequences from the NCBI database and beaver *E. coli* sequences collected from Canada. It remains unclear whether the geographical variability in the reference library may have impacted the classification results. Furthermore, our MST approach involves a complex analysis procedure requiring specialist knowledge, with a long turnaround time to obtain the results, thus, it may not be applicable for use in continuous monitoring programs (Holcomb & Stewart, 2020). This could partly be overcome by screening for library-independent host-specific markers in the water samples, such as *Bacteroides*-based markers. However, while library-independent markers for human, domestic animals (i.e., cows, pigs, and dogs), and bird hosts have been described (Paruch & Paruch, 2022), to our knowledge, such markers have not yet been described for reindeer and beaver hosts, which were of main importance in this study.

Even though the monitoring of eDNA does not directly target fecal contamination, it can be used to map the presence of species that could potentially contribute to fecal contamination in an area, especially given that eDNA has been argued to originate predominantly from fecal matter (Paruch & Paruch, 2022). Analyses using eDNA, however, require specialist training for sampling and downstream analyses, thus precluding its routine implementation for water quality monitoring.

By integrating all three methods (i.e., microbial enumeration, MST, eDNA), the study minimized the individual limitations of each technique and enabled us to achieve a more comprehensive understanding of fecal pollution in our study area. This multifaceted approach, representing the first to our knowledge, enabled us to 1) assess fecal contamination levels in the research area, 2) pinpoint problematic sites in terms of fecal pollution, 3) determine which animal species had the biggest impact at certain sites, 4) attribute a proportion of the contaminating *E.* *coli* isolates to a corresponding host source and 5) clearly distinguish between human and animal pollution, which is particularly important in distinguishing between the basic/natural pollution level caused by wildlife and reindeer and the excessive pollution caused by human activity. This outcome would not have been possible through any single method alone.

## Conclusions

Despite variations in *E. coli* counts across sampling locations and dates, problematic sites regarding fecal pollution were identified in the catchment area of the most upstream part of the Indalsälven River in Jämtland County, Sweden. Microbial source tracking of the collected strains made it possible to identify the original host source (i.e., human, reindeer and/or beaver) of *E. coli* isolates impacting the waters within the sampling area; however, the high proportion of strains that were left unclassified highlights some of the challenges for fecal source tracking, especially when conducted in large catchment areas. The analysis of eDNA further enhances the understanding of fecal pollution sources in cold oligotrophic mountain rivers and can highlight the contribution of unexpected pollution sources that were not targeted in MST. Thus, the integration of microbial enumeration, MST, and eDNA analyses has allowed us to unravel the dominant sources of fecal pollution in the study area and to distinguish between human and animal sources of fecal contamination. Importantly, our results emphasize the need for interdisciplinary approaches for sustainable water management and can support future policy and regulations aimed at protecting sensitive ecosystems in mountain areas.

## Supplementary Information

Below is the link to the electronic supplementary material.

Supplementary file1 (DOCX 63 KB)

Supplementary file2 (XLSX 1566 KB)

## Data Availability

All data generated or analysed during this study are included in this published article and its supplementary information files or available from the authors.
