# Supplementary material for: Unravelling sources of fecal pollution in oligotrophic mountain waters: Integrating Escherichia coli enumeration, microbial source tracking, and eDNA analysis
Source: Environ Monit Assess. 2025 Jul 3;197(8):849. doi: 10.1007/s10661-025-14298-7 (PMC12222391; doi:10.1007/s10661-025-14298-7)
Supplement: Supplementary file 1 — Supplementary file1 (DOCX 63 KB) [file 10661_2025_14298_MOESM1_ESM.docx]

**Unravelling sources of fecal pollution in oligotrophic mountain waters: Integrating *Escherichia coli* enumeration, microbial source tracking and eDNA analysis**

Sharon Maes^a*^, Martin Andersson-Li^b^, Jessica Sjöstedt^c^, Jon Hildahl^d^, Daniel Yu^e^, Norman Neumann^e^, Monica Odlare^a^, Anders Jonsson^a^

^a^Mid Sweden University, Faculty of Science, Technology and Media, Department of Ecotechnology and Sustainable Building Engineering, Akademigatan 1, SE-831 25 Östersund, Sweden

^b^AquaBiota Water Research ABWR AB, Sveavägen 159, SE-113 46 Stockholm, Sweden

^c^MoRe Research Örnsköldsvik AB, Box 70, SE-891 22 Örnsköldsvik, Sweden

^d^Hjortens Laboratorium, Hyggesvägen 21b, SE-831 48 Östersund, Sweden

^e^University of Alberta, School of Public Health, 11328 - 89 Ave NW, Edmonton, Alberta, Canada

^*^Corresponding author: [sharon.maes@hotmail.com](mailto:Sharon.maes@hotmail.com)

Environmental Monitoring and Assessment

# Supplementary data 1: Overview of 60 water samples taken in rivers and tributaries in the research area with information about location, sampling date, water temperature (°C) precipitation (mm), river flow rate (m^3^ s^-1^), storage time (min) between sampling and microbiological analysis, enumeration of *E. coli* (CFU/100mL), Coliforms (CFU/100mL) and Total heterotrophic count (THC, CFU/mL) and eDNA sample number, size (mL) and depth (m)

| **Sampling location** | | **Y-coordinate** | **X-coordinate** | **River basin^1^** | **Catchment area^2^ (km^2^)** | **Date** | **Tourists^3^** | **Water temperature (°C)** | **UV radiation^4^ (µW/cm^2^)** | **Precipitation^5^ (mm)** | **Precipitation^6^** | **River flow rate^7^ (m^3^ s^-1^)** | **River flow rate^8^** |  | **Microbiology** | | | |  | **eDNA** | | |
| --- | --- | --- | --- | --- | --- | --- | --- | --- | --- | --- | --- | --- | --- | --- | --- | --- | --- | --- | --- | --- | --- | --- |
|  |  |  |  |  |  |  |  |  |  |  |  |  |  |  | **Storage time (min)** | ***E. coli* (CFU/100mL)** | **Coliforms (CFU/100mL)** | **THC (CFU/mL)** |  | **Sample nr** | **Sample size (mL)** | **Sample depth (m)** |
| 1 | Handöl village | 63.2605 | 12.43597 | H | 452.35 | 14-Jun-21 | 57 | 8.5 | /^9^ | 24 | High | 48 | High |  | 1990 | 3 | 3 | 128 |  | ENH_02 | 3000 | 1 |
|  |  |  |  |  |  | 29-Jun-21 | 324 | 14 | / | 3 | Low | 6.8 | Low |  | 1935 | 3 | 3 | 109 |  | ENH_09 | 3000 | 1 |
|  |  |  |  |  |  | 07-Jul-21 | 853 | 15.5 | 758 | 5.3 | High | 2.4 | High |  | 1840 | 14 | 17 | 149 |  | ENH_17 | 3000 | 1 |
|  |  |  |  |  |  | 05-Aug-21 | 1510 | 12 | 450 | 0.7 | Low | 1.7 | Low |  | 950 | 3 | 34 | 72 |  | ENH_22 | 3000 | 0.4 |
|  |  |  |  |  |  | 17-Aug-21 | 541 | / | 552 | 0.4 | Low | 22 | Low |  | 1945 | 19 | 200 | 510 |  | ENH_25 | 3000 | 0.8 |
| 2 | Beaver creek | 63.23269 | 12.44803 | H | / | 14-Jun-21 | 57 | 8.5 | / | / | High | / | High |  | 1510 | 6 | 6 | 390 |  | ENH_06 | 2400 | 0.5 |
|  |  |  |  |  |  | 29-Jun-21 | 324 | 14 | / | / | Low | / | Low |  | 1887 | 5 | 18 | 568 |  | ENH_10 | 2400 | 0.6 |
| 3 | Storulvån MS^10^ | 63.16675 | 12.37428 | H | 419.78 | 14-Jun-21 | 853 | 8.5 | 2420 | 24 | High | 47 | High |  | 1435 | 20 | 26 | 770 |  | ENH_08 | 2400 | 0.3 |
|  |  |  |  |  |  | 29-Jun-21 | 1509 | 14 | NA^11^ | 3 | Low | 6.2 | Low |  | 1500 | 4 | 5 | 101 |  | ENH_13 | 3000 | 0.3 |
|  |  |  |  |  |  | 07-Jul-21 | 541 | 16 | 630 | 5.3 | Low | 2.3 | Low |  | 1765 | 16 | 25 | 238 |  | ENH_18 | 3000 | 0.3 |
|  |  |  |  |  |  | 04-Aug-21 | 57 | 13 | / | 0.3 | High | 1.6 | High |  | 1610 | 35 | 120 | 99 |  | ENH_20 | 3000 | 0.2 |
|  |  |  |  |  |  | 17-Aug-21 | 324 | / | / | 0.4 | Low | 19 | Low |  | 1875 | 35 | 190 | 207 |  | ENH_26 | 3000 | 0.8 |
| 4 | Storulvån | 63.16758 | 12.36828 | H | 29.34 | 14-Jun-21 | 853 | 8.5 | 2150 | 20 | High | 2.5 | Low |  | 1470 | 15 | 21 | 242 |  | ENH_07 | 2400 | 0.3 |
|  |  |  |  |  |  | 29-Jun-21 | 1509 | 14 | NA | 3.3 | Low | 0.4 | Low |  | 1440 | 2 | 2 | 125 |  | ENH_14 | 3000 | 0.4 |
|  |  |  |  |  |  | 07-Jul-21 | 541 | 15 | 775 | 5.7 | Low | 0.1 | Low |  | 1730 | 21 | 43 | 161 |  | ENH_19 | 3000 | 0.3 |
|  |  |  |  |  |  | 04-Aug-21 | 57 | 12 | / | 0.2 | High | 0.1 | / |  | 1580 | 20 | 68 | 331 |  | ENH_21 | 3000 | 0.2 |
|  |  |  |  |  |  | 17-Aug-21 | 324 | / | / | 0.4 | Low | 1 | / |  | 1850 | 24 | 85 | 234 |  | ENH_27 | 3000 | 0.4 |
| 5 | Tributary reindeer fence | 63.12042 | 12.41839 | H | 5.27 | 14-Jun-21 | 57 | 8.5 | / | 22 | High | 0.4 | High |  | 1860 | 1 | 1 | 199 |  | ENH_03 | 3000 | 0.3 |
|  |  |  |  |  |  | 07-Jul-21 | 853 | 12 | 289 | / | High | / | Low |  | 2070 | 62 | 73 | 440 |  | ENH_15 | 3000 | 0.2 |

**Supplementary data 1** (continued)

| **Sampling location** | | **Y-coordinate** | **X-coordinate** | **River basin^1^** | **Catchment area^2^ (km^2^)** | **Date** | **Tourists^3^** | **Water temperature (°C)** | **UV radiation^4^ (µW/cm^2^)** | **Precipitation^5^ (mm)** | **Precipitation^6^** | **River flow rate^7^ (m^3^ s^-1^)** | **River flow rate^8^** |  | **Microbiology** | | | |  | **eDNA** | | |
| --- | --- | --- | --- | --- | --- | --- | --- | --- | --- | --- | --- | --- | --- | --- | --- | --- | --- | --- | --- | --- | --- | --- |
|  |  |  |  |  |  |  |  |  |  |  |  |  |  |  | **Storage time (min)** | ***E. coli* (CFU/100mL)** | **Coliforms (CFU/100mL)** | **THC (CFU/mL)** |  | **Sample nr** | **Sample size (mL)** | **Sample depth (m)** |
| 6 | Tjallingån | 63.11303 | 12.45011 | H | 51.16 | 14-Jun-21 | 57 | 8.5 | / | 26 | High | 7.7 | High |  | 1680 | <1 | 2 | 162 |  | ENH_04 | 3000 | 0.5 |
|  |  |  |  |  |  | 29-Jun-21 | 324 | 14 | / | 3.3 | Low | 0.5 | Low |  | 1700 | 1 | 2 | 58 |  | ENH_12 | 3000 | 0.3 |
|  |  |  |  |  |  | 05-Aug-21 | 1510 | 13 | 1600 | 0.8 | Low | 0.1 | Low |  | 810 | 1 | 35 | 54 |  | ENH_23 | 3000 | 0.3 |
|  |  |  |  |  |  | 18-Aug-21 | 254 | 10 | 160 | 29 | High | 3.6 | High |  | 445 | 5 | 44 | 22 |  | ENH_28 | 3000 | 0.3 |
| 7 | Upstream reindeer fence | 63.11242 | 12.4175 | H | 255.82 | 14-Jun-21 | 57 | 8.5 | / | 26 | High | 31 | High |  | 1620 | <1 | <1 | 75 |  | ENH_05 | 3000 | 0.3 |
|  |  |  |  |  |  | 29-Jun-21 | 324 | 14 | / | 2.9 | Low | 4.1 | Low |  | 1760 | <1 | <1 | 64 |  | ENH_11 | 3000 | 0.2 |
|  |  |  |  |  |  | 07-Jul-21 | 853 | 14 | 888 | 5.3 | High | 1.5 | High |  | 1960 | 13 | 14 | 141 |  | ENH_16 | 3000 | 0.3 |
|  |  |  |  |  |  | 05-Aug-21 | 1510 | 15 | 780 | 0.6 | Low | 0.9 | Low |  | 750 | 3 | 17 | 87 |  | ENH_24 | 3000 | 0.3 |
|  |  |  |  |  |  | 18-Aug-21 | 254 | 10 | 160 | 31 | High | 18 | High |  | 385 | 5 | 109 | 18 |  | ENH_29 | 3000 | 0.3 |
| 8 | Downstream Helags MS | 62.91881 | 12.50086 | H | 14.84 | 13-Aug-21 | 764 | 15 | 1730 | 14 | High | 0.6 | High |  | 400 | 10 | 11 | 632 |  | ENE_27 | 3000 | 0.1 |
| 9 | Upstream Helags MS | 62.91264 | 12.50647 | H | 14.84 | 13-Aug-21 | 764 | 14 | 376 | 14 | High | 0.6 | High |  | 355 | 1 | 1 | 41 |  | ENE_28 | 3000 | 0.2 |
| 10 | Enkroken | 63.2645 | 12.22353 | E | 324.64 | 14-Jun-21 | 57 | 8.5 | / | 18 | High | 23 | High |  | 1410 | 15 | 25 | 252 |  | ENE_02 | 3000 | 0.5 |
|  |  |  |  |  |  | 15-Jun-21 | 119 | 7 | / | 4.4 | Low | 38 | High |  | 540 | 33 | 82 | 600 |  | ENE_03 | 2100 | 0.6 |
|  |  |  |  |  |  | 29-Jun-21 | 324 | 14 | / | 3.1 | Low | 4.1 | Low |  | 1365 | 4 | 4 | 215 |  | ENE_07 | 3000 | 0.5 |
|  |  |  |  |  |  | 07-Jul-21 | 853 | 18.5 | 244 | 6.5 | High | 1.9 | Low |  | 1655 | 23 | 33 | 229 |  | ENE_13 | 3000 | 0.2 |
|  |  |  |  |  |  | 04-Aug-21 | 1509 | 13 | NA | 0.2 | Low | 1.3 | Low |  | 1655 | 6 | 25 | 131 |  | ENE_19 | 3000 | 0.2 |
|  |  |  |  |  |  | 17-Aug-21 | 541 | 11 | 789 | 0.4 | Low | 15 | Low |  | 1765 | 155 | 540 | 1360 |  | ENE_29 | 3000 | 0.3 |
| 11 | Downstream Sevedholm | 63.2325 | 12.16169 | E | 274.78 | 15-Jun-21 | 119 | 7 | / | 4.4 | Low | 35 | High |  | 465 | 23 | 70 | 1270 |  | ENE_04 | 2400 | 1 |
|  |  |  |  |  |  | 30-Jun-21 | 885 | 14 | / | 0 | Low | 3.2 | Low |  | 690 | 9 | 22 | 211 |  | ENE_08 | 3000 | 0.3 |
|  |  |  |  |  |  | 07-Jul-21 | 853 | 15.5 | 321 | 6.5 | High | 1.7 | Low |  | 1550 | 31 | 43 | 356 |  | ENE_14 | 3000 | 0.5 |
|  |  |  |  |  |  | 04-Aug-21 | 1509 | 14 | 160 | 0.2 | Low | 1.2 | Low |  | 1760 | 8 | 110 | 193 |  | ENE_22 | 3000 | 0.3 |
|  |  |  |  |  |  | 17-Aug-21 | 541 | 13 | 810 | 0.4 | Low | 14 | Low |  | 1580 | 108 | 550 | 770 |  | ENE_30 | 3000 | 0.3 |

**Supplementary data 1** (continued)

| **Sampling location** | | **Y-coordinate** | **X-coordinate** | **River basin^1^** | **Catchment area^2^ (km^2^)** | **Date** | **Tourists^3^** | **Water temperature (°C)** | **UV radiation^4^ (µW/cm^2^)** | **Precipitation^5^ (mm)** | **Precipitation^6^** | **River flow rate^7^ (m^3^ s^-1^)** | **River flow rate^8^** |  | **Microbiology** | | | |  | **eDNA** | | |
| --- | --- | --- | --- | --- | --- | --- | --- | --- | --- | --- | --- | --- | --- | --- | --- | --- | --- | --- | --- | --- | --- | --- |
|  |  |  |  |  |  |  |  |  |  |  |  |  |  |  | **Storage time (min)** | ***E. coli* (CFU/100mL)** | **Coliforms (CFU/100mL)** | **THC (CFU/mL)** |  | **Sample nr** | **Sample size (mL)** | **Sample depth (m)** |
| 12 | Upstream Sevedholm | 63.23219 | 12.15817 | E | 274.78 | 15-Jun-21 | 119 | 7 | / | 4.4 | Low | 32 | High |  | 420 | 28 | 66 | 530 |  | ENE_05 | 2100 | 1 |
|  |  |  |  |  |  | 30-Jun-21 | 885 | 14 | / | 0 | Low | 3 | Low |  | 640 | 9 | 18 | 215 |  | ENE_09 | 3000 | 0.8 |
| 13 | Tväråbäcken | 63.22472 | 12.17014 | E | 16.19 | 30-Jun-21 | 885 | 14 | / | 0 | Low | 0.1 | Low |  | 570 | 2 | 2 | 131 |  | ENE_10 | 3000 | 0.2 |
|  |  |  |  |  |  | 07-Jul-21 | 853 | 17 | 275 | 6.4 | High | 0.1 | Low |  | 1505 | 210 | 280 | 210 |  | ENE_15 | 3000 | 0.4 |
|  |  |  |  |  |  | 04-Aug-21 | 1509 | 14 | 70 | 0.2 | Low | 0.1 | Low |  | 1710 | 5 | 110 | 330 |  | ENE_23 | 3000 | 0.3 |
|  |  |  |  |  |  | 17-Aug-21 | 541 | 14 | 590 | 0.4 | Low | 0.5 | Low |  | 1560 | 17 | 37 | 239 |  | ENE_31 | 3000 | 0.4 |
| 14 | Creek N Blåhammaren MS | 63.22478 | 12.16533 | E | / | 15-Jun-21 | 119 | 7 | / | 4.6 | Low | 2.4 | / |  | 375 | <1 | 5 | 204 |  | ENE_06 | 2400 | 0.3 |
| 15 | Ranglan | 63.18322 | 12.06764 | E | 43.88 | 30-Jun-21 | 885 | 14 | / | 0 | Low | 0.4 | Low |  | 405 | 77 | 78 | 290 |  | ENE_11 | 2400 | 0.2 |
|  |  |  |  |  |  | 04-Aug-21 | 1509 | 14 | 560 | 0.2 | Low | 0.2 | Low |  | 1995 | 24 | 160 | 199 |  | ENE_21 | 2400 | 0.2 |
|  |  |  |  |  |  | 17-Aug-21 | 541 | 13 | 140 | 0.3 | Low | 1.8 | Low |  | 1345 | 98 | 390 | 1150 |  | ENE_32 | 1500 | 0.3 |
| 16 | Bridge Enan | 63.20064 | 12.09928 | E | 161.78 | 30-Jun-21 | 885 | 14 | / | 0 | Low | 1.8 | Low |  | 340 | 1 | 1 | 65 |  | ENE_12 | 3000 | 0.3 |
|  |  |  |  |  |  | 04-Aug-21 | 1509 | 14 | 775 | 0.2 | Low | 0.7 | Low |  | 2025 | <1 | 11 | 144 |  | ENE_20 | 3000 | 0.2 |
|  |  |  |  |  |  | 17-Aug-21 | 541 | 14 | 33 | 0.4 | Low | 7.2 | Low |  | 1300 | 12 | 45 | 187 |  | ENE_33 | 3000 | 0.4 |
| 17 | Downstream Gamla Sylen | 63.06019 | 12.27531 | E | 27.51 | 08-Jul-21 | 1125 | 14 | 2370 | 3.5 | High | 0.7 | High |  | 560 | <1 | 3 | 38 |  | ENE_16 | 3000 | 0.2 |
|  |  |  |  |  |  | 05-Aug-21 | 1510 | 14 | 610 | 0.6 | Low | 0.3 | Low |  | 530 | <1 | 28 | 61 |  | ENE_24 | 3000 | 0.2 |
| 18 | Downstream Sylarna MS | 63.05228 | 12.2755 | E | 27.51 | 08-Jul-21 | 1125 | 14 | 2520 | 3.5 | High | 0.7 | High |  | 520 | 2 |  | 76 |  | ENE_17 | 3000 | 0.2 |
|  |  |  |  |  |  | 05-Aug-21 | 1510 | 14 | 585 | 0.6 | Low | 0.3 | Low |  | 495 | 1 | 55 | 47 |  | ENE_25 | 3000 | 0.3 |
| 19 | Upstream Sylarna MS | 63.03558 | 12.26375 | E | 27.51 | 08-Jul-21 | 1125 | 14 | 2600 | 3.5 | High | 0.6 | High |  | 480 | 1 |  | 51 |  | ENE_18 | 3000 | 0.1 |
|  |  |  |  |  |  | 05-Aug-21 | 1510 | 14 | 500 | 0.6 | Low | 0.3 | Low |  | 460 | <1 | 74 | 102 |  | ENE_26 | 3000 | 0.4 |

^1^ H: Handölan, E: Enan

^2^ Indicated total catchment area size by SMHI at the corresponding location

^3^ Number of crossings at pedestrian bridge, Storulvån Mountain Station (Data retrieved from the County Administration Board, personal communication, Lansstyrelsen, Tommy Dadell, January 12, 2024)

^4^ Based on the average of the minimum and maximum value of a 1-minute measurement

^5^ The measured precipitation at the nearest SMHI weather station in Storlien-Storvallen on the corresponding date

^6^ Compared to the average precipitation on precipitation days at the SMHI weather station in Storlien-Storvallen of 6.4mm, 2.7mm, and 6.4mm for June, July, and August 2021, respectively

^7^ The modelled flow rate by SMHI at the corresponding location on the corresponding date

^8^ Compared to the average modelled monthly flow rate by SMHI at the corresponding location for the corresponding month

^9^ /: Data is missing

^10^ MS: Mountain Station

^11^ NA: Not applicable, the sun was down at the time of sampling
